# Supplementary material for: Exploratory study of the global intent to accept COVID-19 vaccinations
Source: Commun Med (Lond). 2021 Sep 9;1:30. doi: 10.1038/s43856-021-00027-x (PMC9053214; doi:10.1038/s43856-021-00027-x)
Supplement: Supplementary file 1 — Supplementary Information [file 43856_2021_27_MOESM1_ESM.pdf]

Supplementary information

Supplement to de Figueiredo A & Larson HJ. Global intent to accept COVID-19 vaccinations

Supplementary Methods

A sensitivity analysis is performed to assess the impact of recoding individuals who choose ‘no response / do not know’ to the statement ‘When a vaccine for the coronavirus becomes available, will you get vaccinated?’ (see main text). In total, there are fewer than 100 out of over 26,000 respondents (~0.4%) who respond in this way. The full regression analysis as described in the main text is repeated but (1) removing all respondents who did not provide a response or chose ‘do not know’ (that is, a complete case analysis) [see supplementary figure 1] and (2) recoding all respondents who did not provide a response or chose ‘do not know’ to ‘probably will get vaccinated’ [see supplementary figure 2]. In both cases, the preferred model (via DIC selection) was the intercept-as-outcomes model. There were quantitatively only very small changes to the inferred fixed-effect parameter estimates (compare supplementary figures 1 and 2 with main text figure 2A). With regards to the interpretation of ‘significant results’ there is one fixed-effect parameter which we deem as ‘significant’ in the main text, which become ‘non-significant’ under the sensitivity analysis. (Though we point out that (a) these changes in HPDI are extremely small and (b) the selection of a 95% HPDI to obtain ‘significant’ variables is non-standard in a Bayesian setting). The parameter for ‘no response / do not know’ for whether individuals believe their Government has handled the pandemic well (GOV’T HANDLING) moves from 0.83 (0.68 to 1.00) in the main text to 0.84 (0.69 to 1.02) in the complete case sensitivity and 0.83 (0.68 to 1.01) in the recoding to ‘probably will get vaccinated’. Further, in the main text, the fixed-effect parameter for 55-64-year-olds is 1.13 (0.99 to 1.27), whereas in the complete case analysis this becomes 1.13 (1.00 to 1.27).

	Country or Territory	Methodology	Sample	Coverage	Field Dates
1	ARGENTINA	ONLINE	1016	Nationwide	Nov. 5-19
2	BRAZIL	ONLINE	1120	Nationwide	Nov. 13-20
3	CANADA	ONLINE	1000	Nationwide	Nov. 4-14
4	CHILE	ONLINE	1000	Nationwide	Nov. 6-11
5	CHINA	ONLINE	1000	Nationwide	Nov. 10-16
6	CROATIA	ONLINE	520	Nationwide	Nov. 11-12
7	DENMARK	ONLINE	500	Nationwide	Nov. 1-15
8	ECUADOR	CATI	700	Quito-Guayaquil	Nov. 1-15
9	FINLAND	CAWI	651	Nationwide	Nov. 18-20
10	FRANCE	CAWI	1000	Nationwide	Dec. 8-12
11	GERMANY	ONLINE	1000	Nationwide	Oct. 21-28
12	HONG KONG	ONLINE	509	Nationwide	Nov. 18-23
13	INDIA	ONLINE	500	Nationwide	Nov. 30 - Dec. 4
14	INDONESIA	ONLINE	1000	Nationwide	Nov. 14-25
15	REP. of IRELAND	ONLINE	1001	Nationwide	Nov. 5-10
16	ITALY	ONLINE	1000	Nationwide	Oct. 26-29
17	JAPAN	ONLINE	1137	Nationwide	Nov. 6-9
18	LEBANON	CATI	500	Nationwide	Nov. 5-19
19	MALAYSIA	ONLINE	500	Nationwide	Nov. 1-16
20	MEXICO	ONLINE	500	Nationwide	Nov. 13-23
21	NIGERIA	F2F	1000	Nationwide	Nov. 16-30
22	PAKISTAN	CATI	1103	Nationwide	Nov. 5-15
23	PARAGUAY	CATI	500	Nationwide	Dec. 2-15
24	PERU	ONLINE	1210	Nationwide	Nov. 2-5
25	POLAND	ONLINE	587	Nationwide	Oct. 28-31
26	REP. of KOREA	F2F	1500	Nationwide	Nov. 7-29
27	SERBIA	ONLINE	500	Nationwide	Nov. 12-16
28	SLOVENIA	ONLINE	798	Nationwide	Nov. 11-12
29	SPAIN	ONLINE	1006	Nationwide	Oct. 22-23
30	UK	ONLINE	1000	Nationwide	Dec. 11-13
31	USA	ONLINE	800	Nationwide	Nov. 11-18
32	VIETNAM	TAPI	600	Ha Noi and Ho Chi Minh City	Oct. 31 Nov. 12

Supplementary table 1. **Survey methodologies**
A total of 26,759 individual surveys from 32 countries. Surveys were conducted between October 31, 2020 and December 15, 2020. The data collection type depends on the country being surveyed and the availability and prevalence of, for example, the internet. With the exception of two countries (Ecuador and Vietnam) all surveys are nationwide (see Coverage).

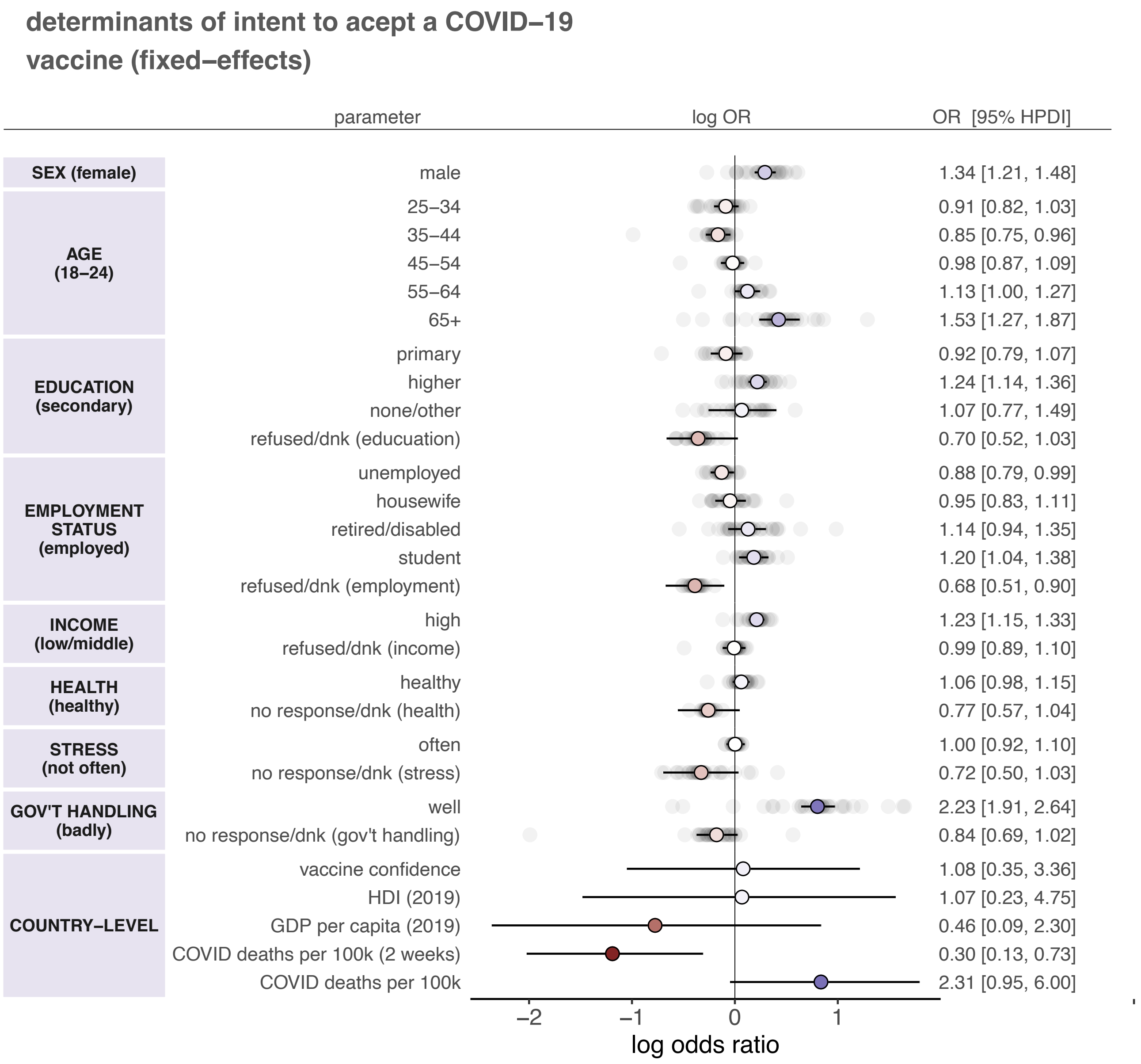

Supplementary Figure 1. **Determinants of intent to accept a COVID-19 vaccine (sensitivity analysis)**. The fixed effect parameters (coloured: reds denote a negative log odds ratio, while blues denote a positive log odds ratio) with 95% highest posterior density intervals (HPDI; black horizontal bars) from the intercepts-as-outcomes model (see main text Methods) which represent an “average” of the effects across all 32 countries (greyed). In this sensitivity analysis, respondents who did not provide a response or who ‘did not know’ are removed from the analysis (see main text, table 2). For each parameter, odds ratios and 95% HPDIs are shown (right panel). The left panel denotes the covariate group with the baseline group shown in parentheses. Quantitatively the results are in excellent agreement with the main text analysis (where those who reply ‘do not know / no response’ are re-coded as ‘probably will not get vaccinated), see main text, figure 2A.

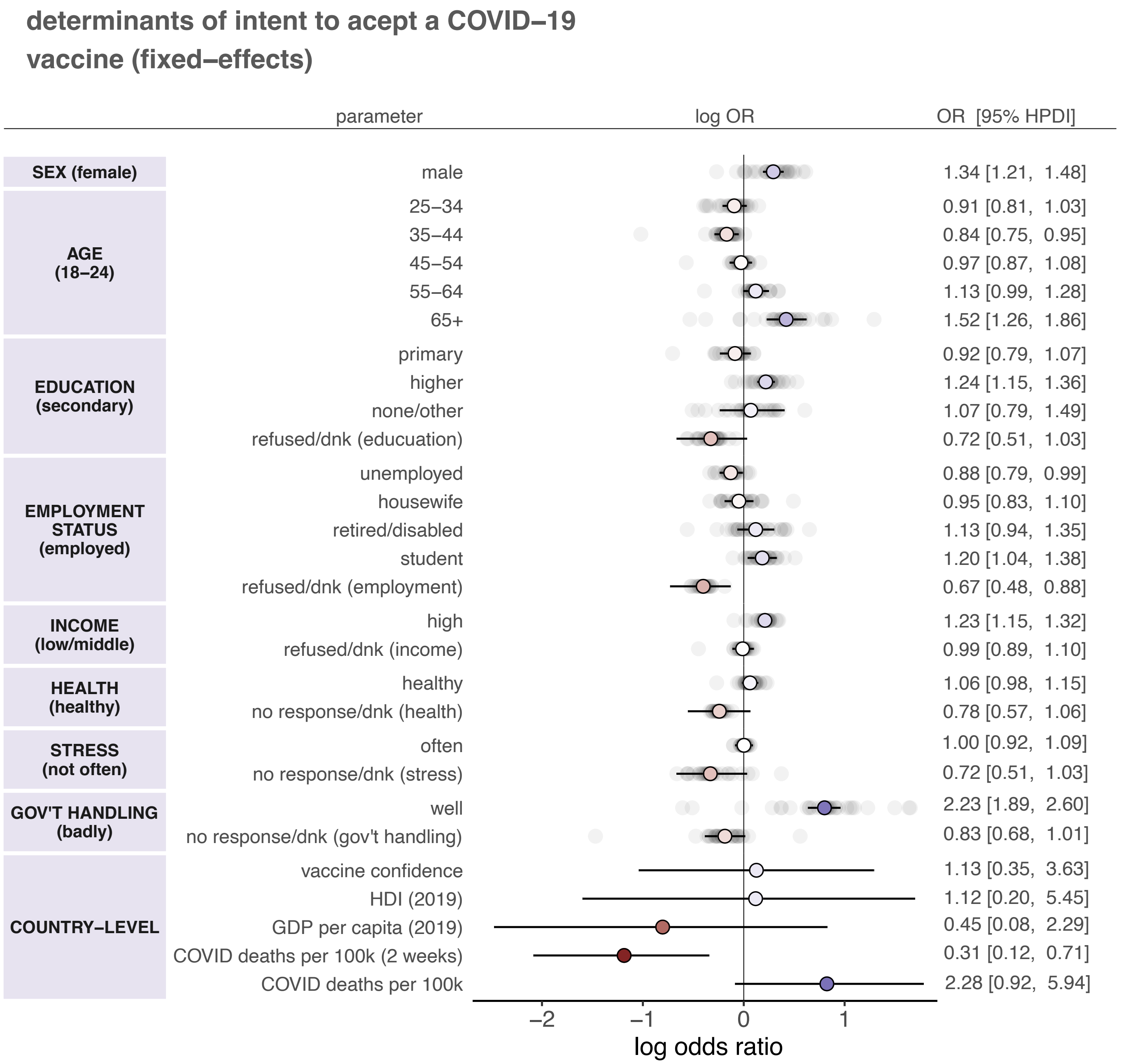

Supplementary Figure 2. **Determinants of intent to accept a COVID-19 vaccine (sensitivity analysis)**. The fixed effect parameters (coloured: reds denote a negative log odds ratio, while blues denote a positive log odds ratio) with 95% highest posterior density intervals (HPDI; black horizontal bars) from the intercepts-as-outcomes model (see main text Methods) which represent an “average” of the effects across all 32 countries (greyed). In this sensitivity analysis, respondents who did not provide a response or who ‘did not know’ are recoded to ‘probably will get vaccinated’ (see main text, table 2). For each parameter, odds ratios and 95% HPDIs are shown (right panel). The left panel denotes the covariate group with the baseline group shown in parentheses. Quantitatively the results are in excellent agreement with the main text analysis (where those who reply ‘do not know / no response’ are re-coded as ‘probably will not get vaccinated), see main text, figure 2A.

Supplementary Notes

The following questions were made available to us from Gallup's End of Year survey.

Health and COVID-19

Q How do you consider your overall health in general?

1. Very healthy
2. Healthy
3. Somewhat unhealthy
4. Unhealthy
9. Do not know / no response

Q How often would you say that you..?

	A lot/ very often	Moderately/ fairly often	A little/ sometimes	Very little/ occasionally	Never	Do not know / no response
Smoke	1	2	3	4	5	9
Drink Alcohol	1	2	3	4	5	9
Exercise	1	2	3	4	5	9
Suffer from stress	1	2	3	4	5	9
Sleep well	1	2	3	4	5	9

Q When a vaccine for the coronavirus will become available, will you get vaccinated?

1. Definitely will get vaccinated
2. Unsure but probably will get vaccinated
3. Unsure but probably won't get vaccinated
4. Definitely won't get vaccinated

Q How would you rate each of the following, with regard to the coronavirus pandemic?

	Very well	Pretty good	Rather badly	Very badly	Do not know
The way your government handled the crisis	1	2	3	4	9
The capacity of the health care system in your country	1	2	3	4	9

Demographics

COL **66.**

D1. Gender

Male 1
Female 2

D2A. Age

Col:	67	68	69	70
Year:				

Refusal *(GO TO D2B)* 0009

COL **71.**

D2B. Age Group

Under 18 1
18 – 24 2
25 – 34 3
35 – 44 4

45 – 54 5
55 – 64 6
65+ 7

ASK INCOME AS YOU NORMALLY WOULD IN YOUR COUNTRY AND THEN RECODE BASED ON THE FOLLOWING CODES.

COL 72.

D3. Income

Low (Bottom quintile/20%) 1
Medium low (Second quintile/20%) 2
Medium (Third quintile/20%) 3
Medium high (Fourth quintile/20%) 4
High (Top quintile/20%) 5
Refused/Don't know/no answer **(CATI&PAPI: DO NOT READ. Online: Show)** 9

ASK EDUCATION AS YOU NORMALLY WOULD IN YOUR COUNTRY AND THEN RECODE BASED ON THE FOLLOWING CODES.

COL 73.

D4. Education: Highest attained

No education/only basic education 1
Completed primary 2
Completed secondary school 3
Completed High level education (University) 4
Completed Higher level of education (Masters, PHD, etc.) 5
Refused/DNK/DNA **(CATI&PAPI: DO NOT READ. Online: Show)** 9

ASK EMPLOYMENT AS YOU NORMALLY WOULD IN YOUR COUNTRY AND THEN RECODE BASED ON THE FOLLOWING CODES.

COL 74.

D5. Employment

Working full (include self-employed) 1
Working Part-time 2
Unemployed 3
Student 4
Housewife 5
Retired/Disabled 6
Refused/DNK/DNA **(CATI&PAPI: DO NOT READ. Online: Show)** 9

Supplementary References

1. Our World In Data. (2021).
2. UNDP. *Human Development Report 2020*. (2020).
3. World Bank: GDP per capita. https://data.worldbank.org/indicator/NY.GDP.PCAP.CD (2021).
4. de Figueiredo, A., Simas, C., Karafillakis, E., Paterson, P. & Larson, H. J. Mapping global trends in vaccine confidence and investigating barriers to vaccine uptake: a large-scale retrospective temporal modelling study. *Lancet* (2020) doi:10.1016/s0140-6736(20)31558-0.
5. Farrar, D. E. & Glauber, R. R. Multicollinearity in Regression Analysis: The Problem Revisited. *Rev. Econ. Stat.* (1967) doi:10.2307/1937887.
6. Snijders, TAB; Bosker, R. *Multilevel analysis: An introduction to basic and advanced multilevel modeling.* (Sage, 2011).
7. Gelman, A. *et al. Bayesian data analysis, third edition*. *Bayesian Data Analysis, Third Edition* (2013).
8. Lazarus, J. V. *et al.* A global survey of potential acceptance of a COVID-19 vaccine. *Nat. Med.* (2020) doi:10.1038/s41591-020-1124-9.
