# Supplementary material for: Exploratory study of the global intent to accept COVID-19 vaccinations
Source: Commun Med (Lond). 2021 Sep 9;1:30. doi: 10.1038/s43856-021-00027-x (PMC9053214; doi:10.1038/s43856-021-00027-x)
Supplement: Supplementary file 5 — Description of Additional Supplementary Files [file 43856_2021_27_MOESM5_ESM.pdf]

Description of Additional Supplementary Files

File Name: Supplementary Data 1

Description: Supplementary Data 1 provides cross tabs for vaccination intent by socio-demographic status for each of the countries used in the study.

File Name: Supplementary Data 2

Description: Supplementary Data 2 provides two excel sheets that contain (a) country codes used in the article and (b) all random-effect (country-specific) parameters from our final intercepts-as-outcomes multilevel model.

File Name: Supplementary Data 3

Description: Supplementary Data 3 contains all raw data that are used to create the manuscript figures.
