# Supplementary material for: Exploratory study of the global intent to accept COVID-19 vaccinations
Source: Commun Med (Lond). 2021 Sep 9;1:30. doi: 10.1038/s43856-021-00027-x (PMC9053214; doi:10.1038/s43856-021-00027-x)
Supplement: Supplementary file 7 — Reporting Summary [file 43856_2021_27_MOESM7_ESM.pdf]

nature research

Corresponding author(s): *Double-blind peer review submissions: write DBPR and your manuscript number here instead of author names.*

Last updated by author(s): *YYYY-MM-DD*

Reporting Summary

Nature Research wishes to improve the reproducibility of the work that we publish. This form provides structure for consistency and transparency in reporting. For further information on Nature Research policies, see our Editorial Policies and the Editorial Policy Checklist.

Statistics

For all statistical analyses, confirm that the following items are present in the figure legend, table legend, main text, or Methods section.

n/a	Confirmed	
☐	☒	The exact sample size (*n*) for each experimental group/condition, given as a discrete number and unit of measurement
☒	☐	A statement on whether measurements were taken from distinct samples or whether the same sample was measured repeatedly
☒	☐	The statistical test(s) used AND whether they are one- or two-sided<br>*Only common tests should be described solely by name; describe more complex techniques in the Methods section.*
☐	☒	A description of all covariates tested
☒	☐	A description of any assumptions or corrections, such as tests of normality and adjustment for multiple comparisons
☐	☒	A full description of the statistical parameters including central tendency (e.g. means) or other basic estimates (e.g. regression coefficient) AND variation (e.g. standard deviation) or associated estimates of uncertainty (e.g. confidence intervals)
☒	☐	For null hypothesis testing, the test statistic (e.g. *F*, *t*, *r*) with confidence intervals, effect sizes, degrees of freedom and *P* value noted<br>*Give P values as exact values whenever suitable.*
☐	☒	For Bayesian analysis, information on the choice of priors and Markov chain Monte Carlo settings
☐	☒	For hierarchical and complex designs, identification of the appropriate level for tests and full reporting of outcomes
☐	☒	Estimates of effect sizes (e.g. Cohen's *d*, Pearson's *r*), indicating how they were calculated

Our web collection on statistics for biologists contains articles on many of the points above.

Software and code

Policy information about availability of computer code

Data collection	n/a
Data analysis	R version 4.0.3 was used for all analysis. JAGS was used with the R package rjags.

For manuscripts utilizing custom algorithms or software that are central to the research but not yet described in published literature, software must be made available to editors and reviewers. We strongly encourage code deposition in a community repository (e.g. GitHub). See the Nature Research guidelines for submitting code & software for further information.

Data

Policy information about availability of data

All manuscripts must include a data availability statement. This statement should provide the following information, where applicable:

- Accession codes, unique identifiers, or web links for publicly available datasets
- A list of figures that have associated raw data
- A description of any restrictions on data availability

All raw WIN World Survey data is available at https://winmr.com/win-world-survey-covid19-vaccine-and-intention-to-travel-in-2021/, while cleaned data used in this study can be found at https://osf.io/8vezs/. All data from Lazarus 2020 is available at https://osf.io/kzq69. The UK data is from a previous large-scale study9. All source data for the article figures can be found in supplementary data 3.

The custom code to implement multilevel ordinal regressions can be found at https://osf.io/8vezs/.

Field-specific reporting

Please select the one below that is the best fit for your research. If you are not sure, read the appropriate sections before making your selection.

☐ Life sciences ☒ Behavioural & social sciences ☐ Ecological, evolutionary & environmental sciences

For a reference copy of the document with all sections, see nature.com/documents/nr-reporting-summary-flat.pdf

Behavioural & social sciences study design

All studies must disclose on these points even when the disclosure is negative.

Study description	Quantitative cross-sectional sample
Research sample	Nationally representative surveys by age, sex, and sub-national region.
Sampling strategy	Survey participants were recruited via online survey panels by ORB (Gallup) International using fixed quotas at the national level for sex, age, and sub-national region.
Data collection	n/a
Timing	Surveys were conducted between September and December 2020
Data exclusions	No data was excluded from the analyses.
Non-participation	Individuals who did not wish to participate simply did not complete the survey. We (the authors) do not have access to this information as ORB (Gallup) International provided a quota-matched sample of respondents.
Randomization	n/a

Reporting for specific materials, systems and methods

We require information from authors about some types of materials, experimental systems and methods used in many studies. Here, indicate whether each material, system or method listed is relevant to your study. If you are not sure if a list item applies to your research, read the appropriate section before selecting a response.

Materials & experimental systems

n/a	Involved in the study	
☒	☐	Antibodies
☒	☐	Eukaryotic cell lines
☒	☐	Palaeontology and archaeology
☒	☐	Animals and other organisms
☐	☒	Human research participants
☒	☐	Clinical data
☒	☐	Dual use research of concern

Methods

n/a	Involved in the study	
☒	☐	ChIP-seq
☒	☐	Flow cytometry
☒	☐	MRI-based neuroimaging

Human research participants

Policy information about studies involving human research participants

Population characteristics	See above
Recruitment	Participants were recruited via an online panel of respondents (see above). There may be technological literacy biases associated with this sample, however, we do control for highest level of education.
Ethics oversight	No ethical approval was required by LSHTM ethics committee for Lazarus 2020 data or Gallup's End of Year survey data as these are both datasets are in the public domain. Ethical approval for the UK study data was obtained by the Imperial College ethics committee on 15 June 2020 with reference 22130.

Note that full information on the approval of the study protocol must also be provided in the manuscript.
